# What can we learn from mixed neuroglial primary cultures about the impact of inflammation on the afferent somatosensory system?

**DOI:** 10.1007/s00424-020-02479-x

**Published:** 2020-10-21

**Authors:** Hans Albert Braun

**Affiliations:** Institute of Physiology and Pathophysiology, Philipps University of Marburg, Deutschhausstrasse 2, 35037 Marburg, Germany

**“Primary culture of the rat spinal dorsal horn: a tool to investigate the effects of inflammatory stimulation on the afferent somatosensory system”**

by Stephan Leisengang et al.

Inflammation strongly impacts the peripheral nociceptive as well as thermoafferent systems. The most pronounced effects of a given inflammatory stimulus on both systems are the induction of fever and the manifestation of inflammatory pain, which is an increased sensitivity of the nociceptive system due to the inflammatory response. Traditionally, the distinct functional components of the afferent somatosensory system have been investigated in vivo in conscious or anesthetized experimental animals. Most frequently, an inflammatory stimulation of the thermoregulatory system to evoke fever was achieved by administration of bacterial lipopolysaccharide (LPS) and continuous recording of body core temperature [5]. Inflammation-associated pain states can be studied by injections of carrageenan into one of the hind paws or ligatures of the sciatic nerve and subsequent assessment of mechanical or heat sensitivities of the stimulated paw [1]. Electrophysiological approaches were also used under in vivo conditions to characterize the properties of peripheral thermosensors [6] or nociceptors [9]. These neurons, which express transient receptor potential (TRP) channels to detect thermal or nociceptive signals, convey their information to neurons located in the superficial dorsal horn of the spinal cord. The sensory processing, especially of the nociceptive system, within the superficial layers of the spinal dorsal horn is complex and involves not only the neurons directly projecting into the brain but also a variety of inhibitory and excitatory interneurons. Electrophysiological investigations of spinal cord tissue slices combined with morphological and neurochemical characterizations seem to provide the most appropriate tools to gain insight into the functional properties of the neuronal circuits within the superficial spinal dorsal horn [7, 8].

In this issue of Pflügers Archiv – European Journal of Physiology, Leisengang et al. [3] introduce and characterize a mixed neuroglial primary culture of the rat superficial dorsal horn. The neurons of this culture show properties which are compatible with those elaborated by use of other experimental approaches. Most of the cultured neurons respond to glutamate, which is released within the superficial dorsal horn as the principle transmitter by the primary afferents of peripheral thermo- and nociceptors. A subpopulation of neurons also responds to substance P, one of the most important peptidergic modulators of nociceptive processing [7]. Finally, spinal thermosensitivity [4] is represented by groups of cultured spinal neurons, which respond to warming or cooling. The disruption of the spinal neuronal network makes the primary culture to a rather artificial system, which is not suitable to characterize neuronal circuits within the superficial dorsal horn. However, the main focus of this study [3] was to characterize the impact of inflammation on the spinal dorsal horn at the cellular level. For this purpose, it is essential that all cell types are present, which naturally occur in the dorsal horn. The mixed primary neuroglial culture established in this study proved to be a useful tool to study possible neuron-astrocyte-microglial interactions upon inflammatory stimulation. A similar culture model was recently introduced from the neonatal rat cortex to study LPS-evoked changes and proved to be an approach to study neuroinflammation in vitro with high accuracy in predicting in vivo neuroinflammatory phenomena [2]. The same properties can be postulated for the neuroglial primary culture of the superficial dorsal horn. One of the major outcomes of this study [3] is the importance of glial cells for an enhanced neuronal glutamate response of LPS-treated cultures, presumably mediated by cytokines. This and other LPS-evoked effects can be used to investigate single cells/cell types and the modulation of their properties under inflammatory conditions. Another possible approach for the neuroglial primary culture is its capacity for drug screening or drug development or testing of substances with putative anti-inflammatory capacities.

## References

[1] Celik MÖ, Labuz D, Keye J, Glauben R, Machelska H (2020). IL-4 induces M2 macrophages to produce sustained analgesia via opioids. JCI Insight.

[2] Goshi N, Morgan RK, Lein PJ, Seker E (2020). A primary neural cell culture model to study neuron, astrocyte, and microglia interactions in neuroinflammation. J Neuroinflammation.

[3] Leisengang S, Nürnberger F, Ott D, Murgott J, Gerstberger R, Rummel C, Roth J (2020) Primary culture of the rat spinal dorsal horn: a tool to investigate the effects of inflammatory stimulation on the afferent somatosensory system. Pflügers Archiv – Eur J Physiol. this issue 10.1007/s00424-020-02478-y PMC7691309 33098464

[4] Pehl U, Simon E, Schmid HA (1997). Properties of spinal neuronal thermosensitivity in vivo and in vitro. Ann N Y Acad Sci.

[5] Roth J, Blatteis CM (2014). Mechanisms of fever production and lysis: lessons from experimental LPS fever. Compr Physiol.

[6] Schäfer K, Braun HA, Kürten L (1988) Analysis of cold and warm receptor activity in vampire bats and mice. Pfügers Arch Eur J Physiol 412:188–194 10.1007/BF00583749 3174381

[7] Todd AJ (2010). Neuronal circuitry for pain processing in the dorsal horn. Nat Rev Neurosci.

[8] Todd AJ (2017). Identifying functional populations among the interneurons in laminae I-III of the spinal cord. Mol Pain.

[9] Zhu YF, Ungard R, Seidlitz E, Zacal N, Huizinga J, Henry JL, Singh G (2016). Differences in electrophysiological properties of functionally identified nociceptive sensory neurons in an animal model of cancer-induced bone pain. Mol Pain.

